# Integrative Analysis of Fungal and Bacterial Microbiomes Across Skin, Blood, and Stool in Rosacea Patients

**DOI:** 10.3390/ijms26178127

**Published:** 2025-08-22

**Authors:** Marie Isolde Joura, Éva Nemes-Nikodém, Antal Jobbágy, Zsuzsanna A Dunai, Nóra Makra, András Bánvölgyi, Norbert Kiss, Miklós Sárdy, Sarolta Eszter Sándor, Péter Holló, Eszter Ostorházi

**Affiliations:** 1 Department of Dermatology, Venerology and Dermatooncology, Semmelweis University, 1085 Budapest, Hungary; joura.marie@phd.semmelweis.hu (M.I.J.);; 2 Károly Rácz Doctoral School of Clinical Medicine, Semmelweis University, 1085 Budapest, Hungary; 3 Institute of Medical Microbiology, Semmelweis University, 1085 Budapest, Hungary; 4 Department of Dermatology, Pál Heim National Institute of Pediatrics, 1089 Budapest, Hungary

**Keywords:** rosacea, internal transcribed spacer, mycobiome, skin, blood, stool, bacterial–fungal interaction

## Abstract

Rosacea is a chronic inflammatory skin disorder with multifactorial pathogenesis involving immune dysregulation and microbial alterations. This study compared the mycobiomes of skin, blood, and stool samples in rosacea patients and healthy controls to assess fungal diversity, abundance, and possible translocation, as well as associations with bacterial microbiomes. Internal transcribed spacer (ITS) region sequencing was performed on samples from 14 rosacea patients and 8 controls. While distinct fungal community compositions were observed across sample types, no significant differences in fungal diversity or genus abundance were found between the patient and control groups in any compartment. *Malassezia* dominated the skin mycobiome, while stool samples showed higher abundances of *Candida* and *Saccharomyces*, which were inversely correlated. Patients with high skin and blood *Malassezia* also exhibited increased *Cutibacterium* abundance, suggesting a potential role in impaired skin barrier integrity. Stool samples with elevated *Saccharomyces* correlated with higher levels of anti-inflammatory bacteria *Prevotella* and *Agathobacter*, whereas *Candida* dominance showed the opposite. These findings suggest that fungal dysbiosis, in the interplay with bacterial communities, may influence rosacea pathogenesis through the gut–skin axis. This work underscores the significance of integrated microbiome research across multiple biological compartments in order to enhance our understanding and potential targeting of microbial factors in rosacea.

## 1. Introduction

Rosacea is a chronic inflammatory skin disorder that predominantly affects the central facial region. It is characterized by persistent erythema, telangiectasia, papules, and pustules. Rosacea is categorized into four clinical subtypes: (1) erythematotelangiectatic rosacea (ETR), (2) papulopustular rosacea (PPR), (3) phymatous rosacea, and (4) ocular rosacea [1]. Despite its high prevalence, particularly among fair-skinned individuals aged 30 to 50, the precise etiology of rosacea remains unclear [2]. Recent findings indicate a multifactorial pathogenesis involving dysregulated immune responses, vascular abnormalities, and neurogenic inflammation [3].

It has been well-documented that the population density of *Demodex* mites residing within the pilosebaceous units of the skin is associated with the pathogenesis and symptomatic severity of rosacea [4]. The physiological conditions of the skin, including the extent of transepidermal water loss and impairment of the skin barrier functions, have been demonstrated to induce alterations in both the quantity and composition of the microbial communities colonizing the skin. A disrupted skin barrier may facilitate increased bacterial colonization, and both barrier dysfunction and microbial colonization can act synergistically to initiate and exacerbate dermatological disorders [5]. The alteration of the skin microbiome in rosacea is closely linked to significant immunological changes that contribute to disease pathogenesis. Dysbacteriosis, defined as an imbalance in bacterial species such as *Cutibacterium* or *Staphylococcus,* results in the upregulation of innate immune receptors like Toll-like receptor 2 (TLR2). This upregulation enhances the production of antimicrobial peptides such as cathelicidins and LL-37. These peptides, when abnormally processed, generate pro-inflammatory fragments that exacerbate symptoms such as erythema and neutrophilic infiltration by perpetuating immune dysregulation and barrier disruption [6,7]. Furthermore, alterations in the gut microbiota have been demonstrated to influence systemic immune responses and cutaneous inflammation via the gut–skin axis. For instance, small intestinal bacterial overgrowth (SIBO) has been reported with increased prevalence in patients diagnosed with rosacea. This condition has the potential to increase intestinal permeability, thereby allowing the translocation of bacterial products and pro-inflammatory cytokines. These factors can trigger cutaneous immune activation [8]. Research investigating the bacterial skin microbiome of individuals diagnosed with rosacea reveals a deviation from the abundance of bacterial genera observed in healthy individuals [9,10,11]. A multitude of studies have identified a correlation between the composition of the enteric bacterial microbiome and rosacea [12,13]. Furthermore, Yun et al. investigated the bacterial microbiome of the blood in patients with rosacea [14]. In a previous study conducted by our research group, we demonstrated that the skin microbiome of rosacea patients differs from that of healthy controls in terms of both alpha and beta diversity, with increased abundances of bacterial genera such as *Staphylococcus, Corynebacterium, Cutibacterium*, and *Neisseria*. However, we did not observe significant genus-level differences in the fecal and blood microbiomes, thus not confirming the transfer of gut microbiome alterations to the skin via the bloodstream. Furthermore, metabolic pathway predictions based on the skin and gut microbiome compositions of rosacea patients indicated the production of pro-inflammatory metabolites, whereas those derived from healthy individuals’ microbiomes were associated with anti-inflammatory biochemical activities [15].

Alterations in the skin mycobiome have been increasingly recognized as important factors in various skin disorders. Research has demonstrated that fungal communities, notably those dominated by the genus *Malassezia*, exhibit substantial alterations in conditions such as atopic dermatitis and seborrheic dermatitis. These changes have been shown to contribute to immune dysregulation and inflammation through mechanisms involving fungal allergens and enzymatic activities that disrupt the skin barrier [16,17]. In contrast, Wang et al. [18] found no significant differences in the skin mycobiome composition between patients with rosacea and healthy controls.

To date, there is a paucity of comprehensive analyses of the mycobiome across multiple biological compartments, including skin, blood, and stool in rosacea patients. In the present study, we aimed to achieve three objectives. 1. We sought to compare the mycobiomes of skin, blood, and stool samples between patients with rosacea and healthy control subjects to determine whether significant differences exist; 2. to assess the fungal abundance across skin, blood, and stool samples in rosacea patients, with the goal of identifying any genera that may translocate from the stool or skin compartments into the bloodstream; 3. and to investigate potential associations between the mycobiome profiles of stool and skin in rosacea patients and the bacterial microbiome previously characterized in our earlier study, in order to elucidate possible links to the pathogenesis of rosacea.

## 2. Results

No detectable DNA was obtained from the extraction negative controls and polymerase chain reaction (PCR) negative controls that were processed concurrently with the samples. These findings indicate the absence of contamination during DNA isolation and internal transcribed spacer (ITS) PCR amplification. The amplification of fungal DNA using the ITS method was unsuccessful in blood samples from four rosacea patients and one healthy control, resulting in insufficient fungal DNA for sequencing. Consequently, all samples (skin, blood and stool) from these five individuals were excluded from subsequent comparative analyses. Adequate sequencing read counts were obtained from stool, blood, and skin samples of 14 rosacea patients and eight control subjects, enabling comparative analysis. The median number of reads per sample, irrespective of sample type, was 20,067 (IQR: 18558).

**Figure 1:**
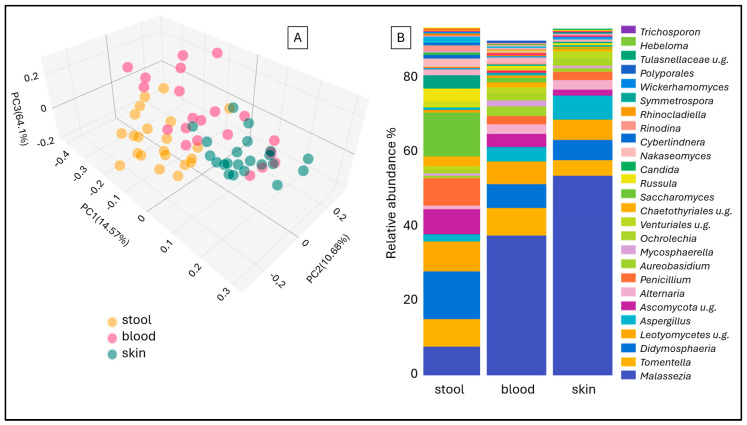
Jaccard beta diversity Principal Coordinate Analysis (PCoA) (**A**) and aggregated stacked bar taxon abundance at genus level (**B**) of stool, blood and skin samples of study participants.

Distinct mycobiome compositions were observed depending on the sample type, irrespective of whether the samples originated from rosacea patients or healthy controls. In the Jaccard beta diversity Principal Coordinate Analysis (PCoA) plot, mycobiome profiles from fecal, blood, and skin samples formed three significantly distinct clusters (Figure 1A). As illustrated by the stacked bar plot, there is a differential abundance of fungi across fecal, blood, and skin samples (Figure 1B). Furthermore, the presence of fungal DNA from all genera detected in blood samples was also noted in stool and skin samples, although generally at intermediate abundance levels in blood. Comparing skin and stool samples of rosacea patients, the genera *Alternaria* (*p* ≤ 0.001), *Aspergillus* (*p* ≤ 0.0001), *Malassezia* (*p* ≤ 0.0001), *Ochrolechia* (*p* ≤ 0.01), and *Venturiales* (*p* ≤ 0.0001) exhibited significantly higher abundances in the skin. Conversely, the stool samples exhibited significantly greater abundances of *Ascomycota* (*p* ≤ 0.0001), *Candida* (*p* ≤ 0.0001), *Chaetothyrales* (*p* ≤ 0.0001), *Didymosphaeria* (*p* ≤ 0.0001), *Penicillium* (*p* ≤ 0.0001), *Rinodina* (*p* ≤ 0.01), *Russula* (*p* ≤ 0.001), and *Saccharomyces* (*p* ≤ 0.001), as well as *Tomentella* (*p* ≤ 0.01). However, the median relative abundances of *Chaetothyrales, Rinodina,* and *Russula* in stool samples were below 5%.

**Figure 2:**
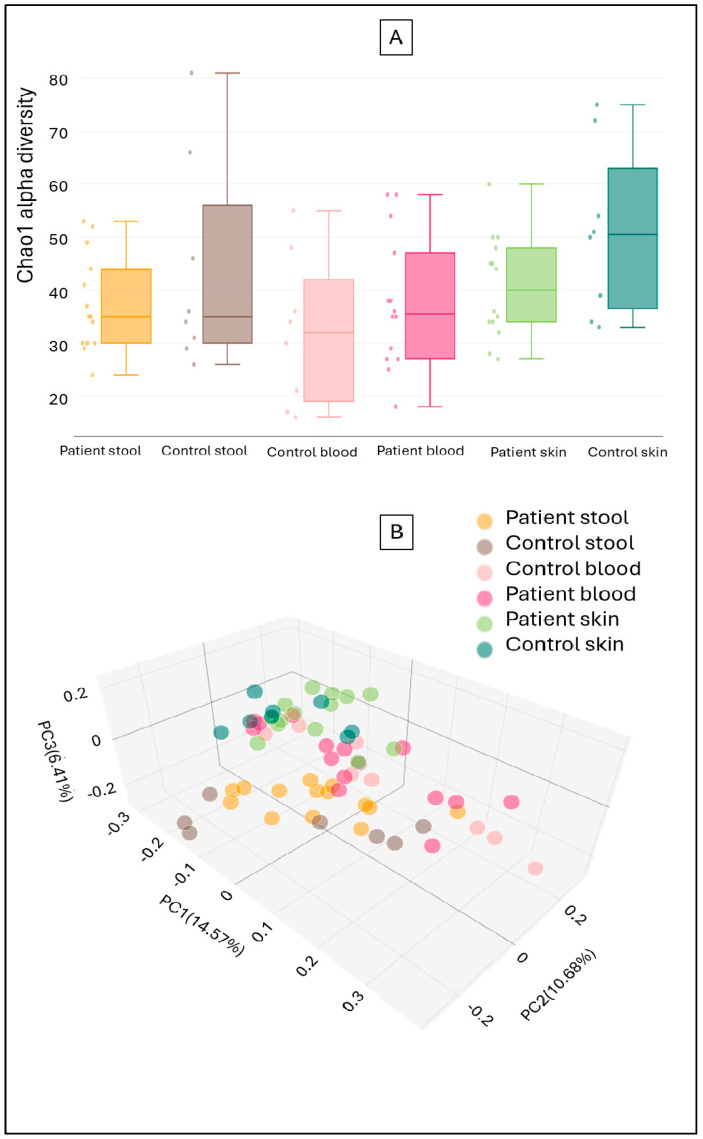
Box plot representation of Chao1 alpha diversity (**A**) and Principal Coordinate Analysis (PCoA) of Jaccard beta diversity (**B**) in fecal, blood, and skin samples from rosacea patients and controls.

No significant differences were found when comparing the alpha abundance of fecal, blood, and skin samples from rosacea patients and healthy controls. This means that a similar number of taxa could be identified in the corresponding sample types regardless of the presence or absence of the disease (Figure 2A). Similarly, no significant differences were observed in the beta diversity of the sample pairs, indicating no significant differences in fungal composition or relative abundance (Figure 2B). Comparative analysis of samples from rosacea patients and healthy controls revealed no fungal genera with significantly different abundances in the skin, blood, or stool mycobiomes.

**Figure 3:**
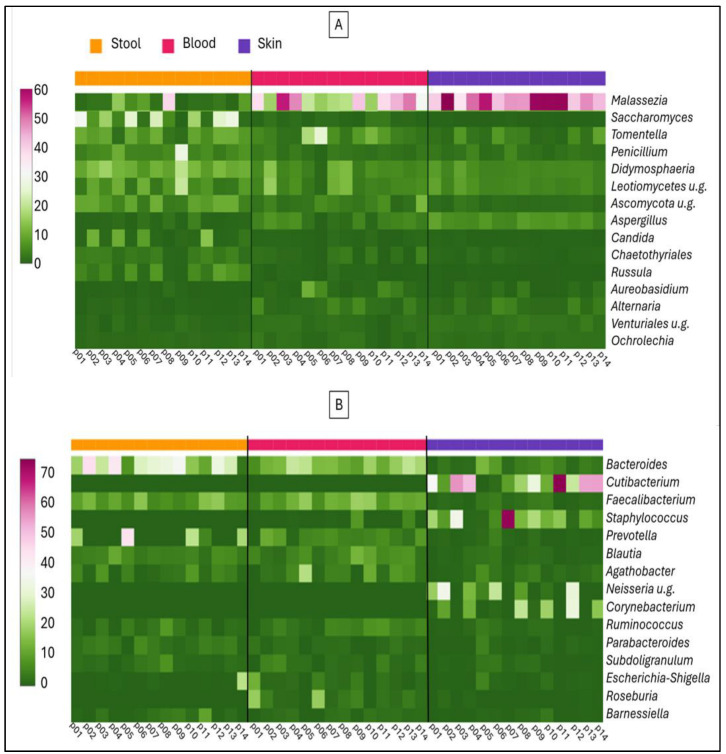
Heatmap presentation of the 15 most prevalent fungal (**A**) and bacterial (**B**) genera detected in stool, blood, and skin samples from 14 rosacea patients (p01–p14).

Fungal DNA can enter the bloodstream from various body surfaces, including oral mucosa, genitalia, urinary tract, skin, and gastrointestinal mucosa. In the present study, we compared the blood microbiome composition exclusively with the latter two sources. As shown in Figure 3, we did not identify any fungal taxa whose DNA presence in the blood could be conclusively attributed to stool alone. In the stool samples, the abundances of *Saccharomyces* and *Candida* were inversely correlated. The Figure 3A heatmap shows that the *Malassezia* genus was present at over 40% abundance in all skin samples, however, this fungal DNA was only detected at appreciable levels in a few corresponding blood samples. Bacterial taxa that constitute the blood microbiome can also originate from multiple anatomical sites within the body. Figure 3B shows that bacterial genera characteristic of the stool are detectable in the blood at appreciable levels. Conversely, bacterial genera associated with the skin (e.g., *Corynebacterium, Cutibacterium, Neisseria*, and *Staphylococcus*) are present in the blood at low abundance and cannot detect above background levels alongside other bacterial taxa.

**Figure 4:**
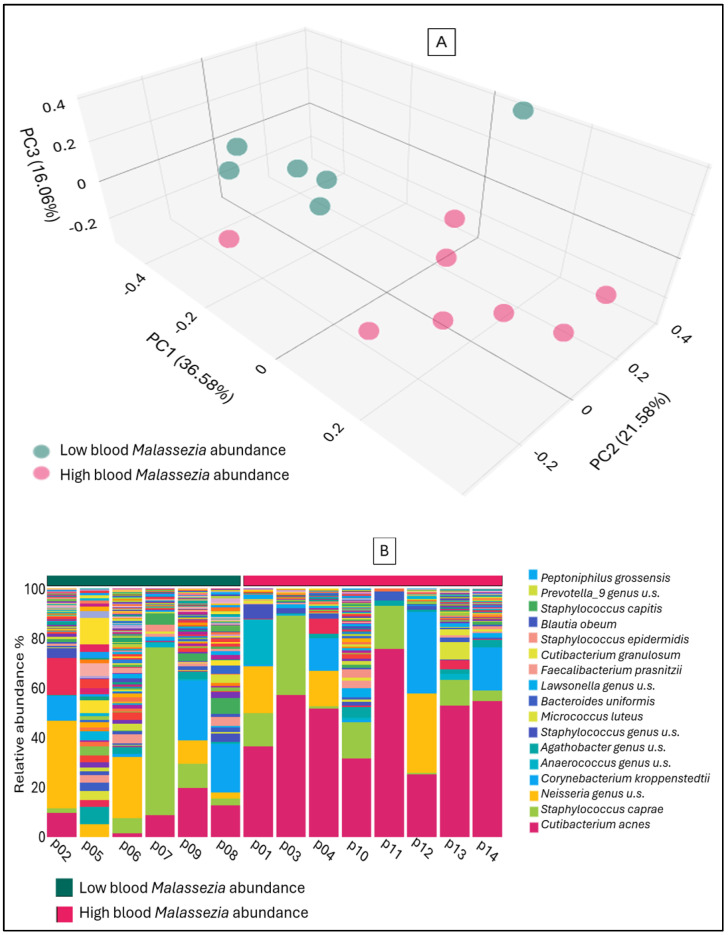
Principal Coordinate Analysis (PCoA) of Jaccard beta diversity (**A**) and stacked bar bacterial abundance (**B**) in skin samples of rosacea patients according to blood *Malassezia* abundance.

A high abundance of the genus *Malassezia* was observed in all skin samples; however, fungal DNA was detected in the bloodstream in only a subset of cases. This raises the question of whether the bacterial community composition associated with the fungi on the skin influences the translocation of fungal DNA into the blood, and whether it affects skin integrity and permeability. As illustrated in Figure 4, the composition of the skin bacterial microbiome, with samples grouped according to whether the genus *Malassezia* is detected in the blood at high or low abundance. The significant difference in beta diversity (*p* = 0.001) between the two groups is justified by the evident variations in bacterial abundances. The group with high *Malassezia* abundance in the blood exhibits a significantly greater abundance of *Cutibacterium* (*p* = 0.002) on the skin. The skin of the subjects with low *Malassezia* abundance in the blood exhibited a significantly higher abundance of the genera *Faecalibacterium* (*p* = 0.02) and *Prevotella* (*p* = 0.01).

Stool samples from rosacea patients demonstrated a significantly higher median relative abundance of multiple fungal genera compared to skin samples, including *Ascomycota, Candida, Chaetothyrales, Didymosphaeria, Penicillium, Rinodina, Russula, Saccharomyces*, and *Tomentella*. However, notable differences emerged in the distribution of these fungi across biological compartments: *Ascomycota, Didymosphaeria*, and *Tomentella* exhibited greater relative abundance in blood samples than in stool, implying that their DNA may originate from sources other than, or in addition to, the gastrointestinal tract. Moreover, the median relative abundances of *Chaetothyrales, Rinodina,* and *Russula* in stool were consistently below 5%, indicating a limited presence.

In contrast, *Saccharomyces* and *Candida* were among the most abundant genera detected in stool samples and are well-established as relevant to human mycobiome dynamics. Taken together, these data justify focusing subsequent analyses exclusively on *Saccharomyces* and *Candida*, as they represent the most pertinent fungal taxa for exploring potential links between the gut mycobiome and rosacea pathogenesis.

**Figure 5:**
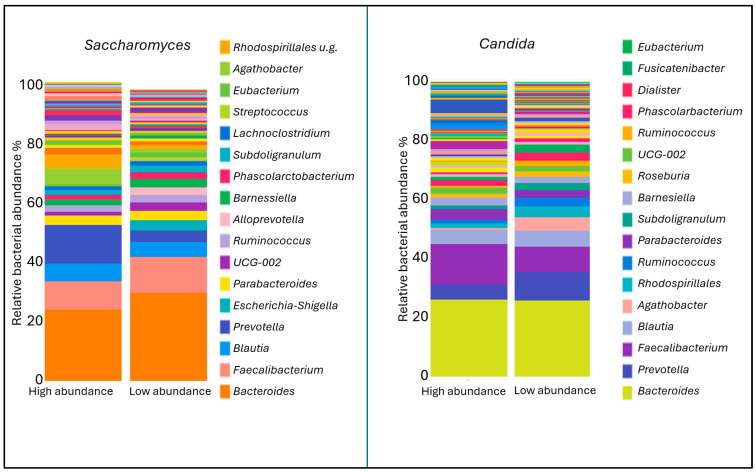
The composition of the bacterial microbiome in groups with high and low *Saccharomyces* or *Candida* abundance shown in stacked bar charts.

**Figure 6:**
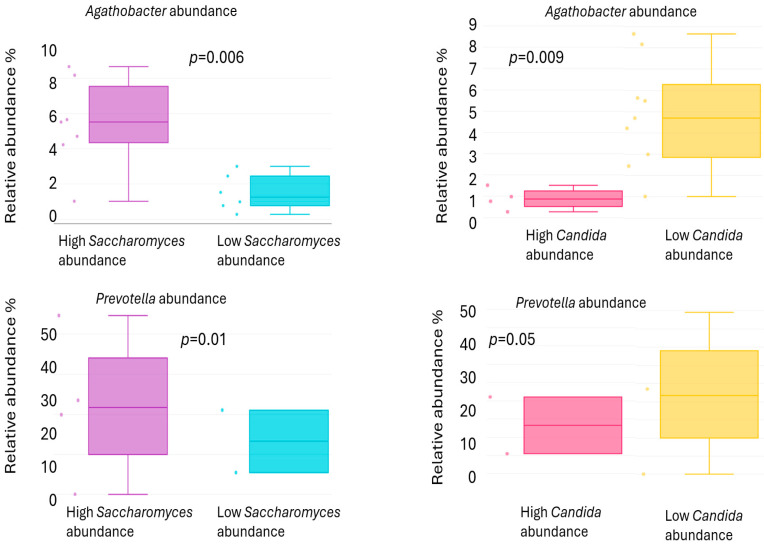
The abundance of *Agathobacter* and *Prevotella* in the stool of rosacea patients depends on the abundance of *Saccharomyces* and *Candida*.

In the stool samples of rosacea patients, *Candida* exhibited a higher median relative abundance, while *Saccharomyces* showed a lower median relative abundance compared to healthy controls; however, these differences were not statistically significant. Figure 5 shows the abundances of bacterial genera associated with the two fungal genera that appear in opposite quantities in the stool. In the group with high *Saccharomyces* abundance, the levels of *Prevotella* (*p* = 0.01) and *Agathobacter* (*p* = 0.006) were found to be significantly higher. Conversely, these two bacterial genera were present in greater abundance in the group with low *Candida* abundance (Figure 6).

## 3. Discussion

Fungal dysbiosis plays a critical role in the pathogenesis of inflammatory skin diseases by disrupting the delicate balance of the skin microbiota and provoking immune dysregulation. Numerous studies have demonstrated that in conditions such as atopic dermatitis, seborrheic dermatitis, or psoriasis, alterations in the fungal community are associated with increased fungal diversity and overgrowth, which exacerbate skin inflammation [16,19,20,21,22]. A comparison of rosacea patients and healthy controls has thus far revealed significant disparities in bacterial alpha and beta diversity, as well as in the abundance of bacterial genera. However, no significant differences were observed between the rosacea and control groups in either fungal microbiome diversity or abundances of fungal genera in skin samples [18].

While the roles of bacteria have been the subject of more extensive research, the specific contributions of the gut mycobiome in skin disorders remain to be fully elucidated. Scientific studies have already reported discrepancies in the gut mycobiome between patients with psoriasis [23] and atopic dermatitis [24,25,26] in comparison to healthy controls. Nevertheless, to date, no publications have addressed this aspect in rosacea. The present study offers novel insights into the mycobiome composition across multiple biological compartments—skin, blood, and stool—in patients with rosacea.

Regardless of whether samples were obtained from healthy individuals or rosacea patients, analysis of skin, blood, and stool specimens revealed significant differences in both beta diversity and fungal abundances across the mycobiomes of the three distinct compartments. All fungal genera detected in the blood were also present in the mycobiome composition of either the skin or stool. However, these fungi may have originated not only from these two sites but also from the oral cavity, respiratory tract, or urogenital mucosa [27]. In patients diagnosed with rosacea, the most significant disparities in the gut and skin mycobiome were characterized by a considerably elevated abundance of *Malassezia* on the skin, and higher abundances of *Saccharomyces* and *Candida* in the stool samples. According to findings of the Human Microbiome Project data [28], four fungal genera—*Saccharomyces*, *Malassezia, Candida*, and *Cyberlindnera*—have been identified as predominant in the human gut mycobiome. However, the gut mycobiome demonstrates considerable variability both interindividually and intraindividually over time, suggesting a lower degree of stability in comparison to the bacterial microbiome. This instability is partially attributed to fungi introduced from environmental sources and diet, as many fungal genera detected in fecal samples are likely transient and unable to persist under gut conditions such as temperature, pH, and low oxygen levels. Supporting evidence shows that *Saccharomyces* abundance in feces correlates with dietary intake, while *Candida* presence relates to oral hygiene [29].

Comparison of alpha diversity in fecal, blood, and skin samples from patients with rosacea and healthy controls revealed no statistically significant differences, indicating that a comparable number of fungal taxa were present across the respective sample types irrespective of disease status. Likewise, analysis of beta diversity showed no significant variation between the groups, suggesting that neither the composition nor the relative abundance of fungal communities differed markedly in the examined sample types. Consistent with the findings of Wang et al. [18], we did not observe significant differences in fungal genus abundances between rosacea patients and healthy controls in skin samples. Additionally, we extended the analysis to include blood and stool samples from both groups, and similarly, no significant differences in fungal abundances were detected in these compartments.

It is understandable that the relative abundance of fungal genera constituting the blood mycobiome does not directly mirror the composition of the skin or fecal mycobiomes, nor is it simply an average of these two compartments, as fungal DNA originating from the oral cavity, respiratory tract, and urogenital tract can alter the proportional representation. Notably, in the blood samples analyzed in our study, whenever a substantial fungal genus abundance (exceeding 30%) was detected, it was predominantly *Malassezia*. The genus *Malassezia* was identified at a frequency of over 40% obtained from patients diagnosed with rosacea. However, its fungal DNA was detected at significant levels in only a few of the blood samples that were obtained from these patients. In our previous study, we found that, in comparison with healthy controls, the skin of patients with rosacea exhibited higher abundances of *Cutibacterium, Staphylococcus*, *Corynebacterium*, and *Neisseria*. The differences observed for the latter two genera were found to be statistically significant. Conversely, the skin of the healthy control group exhibit significantly higher abundances of *Faecalibacterium, Prevotella, Ruminococcus*, and *Subdoligranulum* [15]. When rosacea patients were divided into two groups based on whether *Malassezia*, which was consistently present on their skin at over 40% relative abundance, also exhibited high abundance in their blood, significant differences were confirmed in the composition of their skin microbiome. Patients who exhibited significantly higher abundances of *Cutibacterium* alongside *Malassezia* on their skin showed greater translocation of *Malassezia* into their blood mycobiome. In contrast, patients whose blood contained only low levels of *Malassezia* tended to have fungal communities associated with *Faecalibacterium* and *Prevotella*, genera more commonly found on healthy skin. The role of *Cutibacterium* in the development of rosacea and its immunomodulatory effects appears to be complex and somewhat contradictory. While *Cutibacterium acnes subsp. defendens* exhibits anti-inflammatory properties that may alleviate rosacea-like inflammation [30], other *C. acnes* strains, particularly when associated with *Staphylococcus*, are thought to contribute to the inflammatory processes underlying rosacea pathogenesis [31]. The results of our current study suggest that the *Cutibacterium* strains constituting the microbiome of our rosacea patients, together with *Malassezia* fungi, may have played a role in the impairment of skin integrity.

A comparison of stool samples from individuals diagnosed with rosacea and skin samples from these patients revealed a statistically significant higher median relative abundance of certain genera. These genera include: *Ascomycota, Candida*, *Chaetothyrales, Didymosphaeria, Penicillium, Rinodina, Russula, Saccharomyces*, and *Tomentella*. Among these genera, *Ascomycota, Didymosphaeria*, and *Tomentella* exhibited higher relative abundance values in the blood than in the stool, suggesting that the DNA of these fungi entered the bloodstream not only from the stool or possibly from other sources. The median relative abundances of *Chaetothyrales, Rinodina*, and *Russula* in stool samples were below 5%. In light of the aforementioned considerations, it was determined that *Saccharomyces* and *Candida*, two fungal genera that have been identified in the stool sample, were of particular significance. Consequently, a more in-depth investigation was undertaken to ascertain the role of these genera in the context of rosacea. A comparative analysis of stool samples from patients diagnosed with rosacea and healthy individuals revealed a higher median relative abundance of *Candida*, with a concomitant decrease in *Saccharomyces*, although these differences did not attain statistical significance. Research in animal models indicates that *Saccharomyces cerevisiae* can both exacerbate and attenuate gut inflammation, depending on the context and strain [32,33]. Some studies report that *S. cerevisiae* colonization may exert a protective effect on gut health [34], while other studies demonstrate that it can exacerbate colitis by affecting gut barrier permeability and host metabolism [35]. Furthermore, an imbalance in the composition of fungal communities, manifested as a decrease in *S. cerevisiae* and an increase in *Candida* species, has been observed in patients suffering from Crohn disease. These findings establish a correlation between alterations in gut mycobiota and the severity of disease symptoms, as well as the occurrence of “leaky gut” syndrome [36]. A subsequent examination of the relationship between the fungal composition of the stool and the presence of associated bacteria revealed that stools with a high abundance of *Saccharomyces* had a significantly higher abundance of the bacterial genera *Prevotella* and *Agathobacter,* whereas stools dominated by *Candida* showed a significantly lower abundance of these bacteria. In our previous study on the relationship between rosacea and *Prevotella*, we found that the bacterial genus abundance was significantly higher on the skin of healthy individuals [15]. A body of research has examined the relationship between inflammatory skin disorders and the composition of the gut microbiome. This research has found that a higher abundance of certain *Prevotella* strains reduced allergic skin inflammation in atopic dermatitis and decreased inflammation in psoriasis [37,38]. A bi-directional two-sample Mendelian randomization study confirmed that a higher abundance of *Prevotella* in the gut microbiome was associated with a decreased risk of rosacea [39]. A paucity of direct evidence exists that specifically links *Agathobacter* to individual inflammatory skin diseases. However, its depletion in systemic inflammatory conditions, as well as its key role in producing anti-inflammatory metabolites like butyrate, suggest that it could be a beneficial genus for maintaining gut, and consequently, skin immune homeostasis [40]. While the difference was not statistically significant, *Saccharomyces* abundance was higher in healthy gut flora, whereas *Candida* abundance was more pronounced in patients with rosacea patient. The positive or negative associations of anti-inflammatory bacteria with these two fungal genera further support the hypothesis that the fungal composition of the stool also influences the pathomechanism of rosacea.

Despite the encouraging results of this study, it is imperative to acknowledge the inherent limitations that must be taken into account when interpreting the findings. The cross-sectional design is known to impose limitations on the extent to which causal inferences can be made. Furthermore, the limited sample size may hinder the generalizability of the results obtained. It is important to note that the microbiome exhibits significant variation among different populations, and the present study only included a small number of rosacea patients from a single country. It is imperative to acknowledge this geographic and demographic restriction as a significant limitation. Moreover, the intricacies inherent in fungal taxonomy and the difficulties in accurately profiling low-biomass samples such as blood necessitate meticulous interpretation. A limitation of the present study is that it focused solely on the relationship between bacterial and fungal microbiome composition and rosacea. A potential avenue for further research is to investigate the presence and abundance of *Demodex* mites in relation to these factors. In order validate and expand upon those observations, longitudinal studies with larger cohorts and functional analyses are essential.

In conclusion, our study underscores the significance of the mycobiome in rosacea, emphasizing its potential role not only at the local skin level but also systemically. These findings lay the foundation for integrated microbiome research that encompasses multiple microbial kingdoms and biological compartments, thereby facilitating a more profound comprehension of rosacea pathogenesis and informing the development of novel diagnostic and therapeutic strategies.

## 4. Materials and Methods

### 4.1. Sample Collection

This study conducted a molecular microbiome analysis to compare stool, blood, and skin samples obtained from 14 patients diagnosed with rosacea and eight healthy control subjects. Recruitment occurred between February and August 2021 at the Outpatient Clinic of the Department of Dermatology, Venereology, and Dermatooncology at Semmelweis University, Budapest, Hungary. Only patients newly diagnosed with rosacea and not previously treated were included. Inclusion criteria required a first-time diagnosis of rosacea, while exclusion criteria encompassed pregnancy, use of probiotics or antibiotics within the preceding six months, any gastrointestinal disorders or symptoms in the last four weeks, and any topical or systemic treatment targeting skin conditions within the past four weeks. The control group consisted of eight healthy volunteers matched for age and sex distribution, recruited concurrently. Healthy controls without any dermatological conditions were included in the study, and the exclusion criteria for the control group were identical to those applied to the rosacea patients. Detailed demographic and clinical characteristics of all participants are summarized in Table 1**.**

A minimum of 3 mL of whole blood was drawn into citrate-containing VACUETTE collection tubes (Greiner Bio-One, Stonehouse, UK). Fecal and skin swab specimens were collected using Zymo DNA/RNA Shield tubes (Zymo Research Corp., Irvine, USA). Participants were instructed to refrain from washing the bilateral cheek areas for 24 h prior to skin sample collection. Skin swabs were collected from both cheeks using sterile foam-tipped swabs, with each area swabbed vigorously for 30 s while rotating the swab to ensure adequate sample acquisition. All specimens were stored at −80 °C until DNA extraction.

### 4.2. DNA Isolation

The extraction of the DNA from skin and stool samples was conducted using the ZymoBIOMICS DNA Miniprep Kit (Zymo Research Corp., Irvine, CA, USA). For the extraction of blood samples, the protocol of the NucleoSpin Blood Mini Kit (Macherey-Nagel, Allentown, PA, USA) was followed, in accordance with the manufacturer’s guidelines.

### 4.3. ITS Mycobiome Analysis

The conventional Illumina fungal metagenomic protocol was adapted to facilitate the analysis of the ITS mycobiota. The PCR conditions were optimized by increasing the input of purified DNA to 6.25 μL per reaction and reducing the primer volume to 3 μL to limit primer dimer formation. The amplification cycles were extended to 30 cycles to ensure sufficient target region amplification. To eliminate non-specific amplification products and primer dimers, a two-step purification process was employed using sequential bead cleanups of 25 μL and 10 μL with QuantaBio SparQ PureMag Beads (QIAGEN, Germantown, MD, USA), effectively enriching the desired amplicons. All experimental procedures were performed in duplicate to minimize contamination and enhance reproducibility. Negative controls for both DNA extraction and PCR amplification were included in each batch to monitor for potential reagent-derived contamination. The quality and size distribution of the PCR libraries were evaluated using the Agilent 2100 Bioanalyzer with the DNA 1000 Kit (Agilent Technologies, Waldbronn, Germany). Libraries were normalized to equimolar concentrations, pooled, and subsequently sequenced on an Illumina MiSeq platform (Illumina, San Diego, CA, USA) using the MiSeq Reagent Kit v3 (600-cycle paired-end). The raw sequence data were retrieved via Illumina BaseSpace and subsequently analyzed through the CosmosID bioinformatics platform (CosmosID Inc., Germantown, MD, USA).

### 4.4. Statistical Analysis

Comparative statistical analyses between sample cohorts were conducted using the Wilcoxon Rank Sum test to assess Chao1 alpha diversity, and PERMANOVA was applied to Jaccard distance matrices derived from Principal Coordinates Analysis (PCoA) for evaluating beta diversity. These analyses were performed using the statistical tools integrated within the CosmosID bioinformatics platform. The statistical significance was established at a two-tailed *p*-value threshold of ≤0.05.

The correlation between the fungal and bacterial microbiomes present in the samples was also analyzed using the CosmosID bioinformatics pipeline. For the bacterial microbiome data, we employed sequencing datasets that had been previously generated [15], which were then reanalyzed from a novel perspective through the application of updated bioinformatics methods.

### 4.5. Ethical Considerations

The protocol for sample collection was reviewed and endorsed by the Ethics Committee of Semmelweis University (SE RKEB: 282/2020). This study was conducted in full compliance with the ethical principles outlined in the Declaration of Helsinki, thereby ensuring respect for and the protection of all human participants. Written informed consent was obtained from all participants, permitting the publication of data derived from their personal test results. In order to maintain confidentiality of the data, the results of this study were presented in a manner that anonymized the information, thereby preventing any possibility of identifying individual subjects.

## 5. Conclusions

Fungal dysbiosis plays a significant role in the pathogenesis of inflammatory skin diseases by disrupting the balance of microbes and provoking immune dysregulation. While rosacea patients do not show significant differences in fungal diversity or genus abundance on the skin, blood, or stool samples compared to healthy controls, elevated levels of *Malassezia* on the skin and altered gut fungal profiles—characterized by increased *Candida* and reduced *Saccharomyces* abundances—suggest fungal involvement in the pathophysiology of rosacea. Furthermore, the correlations between these fungal genera and anti-inflammatory bacterial taxa, such as *Prevotella* and *Agathobacter,* emphasize the complex interplay between the gut mycobiome and bacterial communities in modulating skin inflammation. Importantly, *Cutibacterium* species, which were found in higher abundance alongside *Malassezia* in rosacea skin, likely contribute to impaired skin barrier integrity, thereby exacerbating inflammatory processes. These findings emphasize the importance of the gut–skin axis and the fungal microbiome in rosacea and other inflammatory skin disorders, and highlight the need for further research into targeted microbiome-based therapies.

## Figures and Tables

**Table 1:** Demographic and clinical characteristics of the study participants, including sample size, gender distribution, median age (years) with interquartile range (IQR), and rosacea subtype classification.

	Rosacea Patients (14)	Healthy Controls (8)
Gender: male/female	4/10	2/6
Age, Year (median + IQR)	48.5, IQR:25	41, IQR: 27
Diagnosis	13 PPR, 1 PPR + ETR	-
BMI	27, IQR: 7	26, IQR: 6.5
Consumption of alcohol: yes/no	7/7	4/4
Smoking: yes/no	6/8	2/6
Diet related symptom exacerbation: yes/no	8/6	-

## Data Availability

The datasets generated and analyzed during the current study are available in the SRA repository: SRA/PRJNA 1189573/https://www.ncbi.nlm.nih.gov (accessed on 22 November 2024) and SRA/PRJNA PRJNA1288008/https://www.ncbi.nlm.nih.gov (accessed on 8 July 2025).

## Abbreviations

The following abbreviations are used in this manuscript:

ETR	Erythematotelangiectatic rosacea
ITS	Internal transcribed spacer
IQR	interquartile range
PCoA	Principal Coordinate Analysis
PCR	Polymerase Chain Reaction
PPR	Papulopustular rosacea
SIBO	Small Intestine Bacterial Overgrowth
TLR	Toll-like Receptor

## References

[1] Wilkin J., Dahl M., Detmar M., Drake L., Feinstein A., Odom R., Powell F. (2002). Standard classification of rosacea: Report of the national rosacea society expert committee on the classification and staging of rosacea. J Am. Acad. Dermatol..

[2] Searle T., Al-Niaimi F., Ali F.R. (2021). Rosacea. Br. J. Hosp. Med..

[3] Aedo G., Chahuan M., Gatica E., Herrera I., Parada L.F., Seguel A., Murray N.P., Aedo S., Aragon-Caqueo D. (2025). Managing a Burning Face: Clinical Manifestations and Therapeutic Approaches for Neurogenic Rosacea. Int. J. Mol. Sci..

[4] Moran E.M., Foley R., Powell F.C. (2017). Demodex and rosacea revisited. Clin. Dermatol..

[5] Yuan C., Ma Y., Wang Y., Wang X., Qian C., Hocquet D., Zheng S., Mac-Mary S., Humbert P. (2020). Rosacea is associated with conjoined interactions between physical barrier of the skin and microorganisms: A pilot study. J. Clin. Lab. Anal..

[6] Mylonas A., Hawerkamp H.C., Wang Y., Chen J., Messina F., Demaria O., Meller S., Homey B., Di Domizio J., Mazzolai L. (2023). Type I IFNs link skin-associated dysbiotic commensal bacteria to pathogenic inflammation and angiogenesis in rosacea. JCI. Insight..

[7] Qi X., Xiao Y., Zhang X., Zhu Z., Zhang H., Wei J., Zhao Z., Li J., Chen T. (2024). Probiotics suppress LL37 generated rosacea-like skin inflammation by modulating the TLR2/MyD88/NF-kappaB signaling pathway. Food. Funct..

[8] Li J., Yang F., Liu Y., Jiang X. (2024). Causal relationship between gut microbiota and rosacea: A two-sample Mendelian randomization study. Front. Med..

[9] Zaidi A.K., Spaunhurst K., Sprockett D., Thomason Y., Mann M.W., Fu P., Ammons C., Gerstenblith M., Tuttle M.S., Popkin D.L. (2018). Characterization of the facial microbiome in twins discordant for rosacea. Exp. Dermatol..

[10] Thompson K.G., Rainer B.M., Antonescu C., Florea L., Mongodin E.F., Kang S., Chien A.L. (2020). Comparison of the skin microbiota in acne and rosacea. Exp. Dermatol..

[11] Rainer B.M., Thompson K.G., Antonescu C., Florea L., Mongodin E.F., Bui J., Fischer A.H., Pasieka H.B., Garza L.A., Kang S. (2020). Characterization and analysis of the skin microbiota in rosacea: A case-control study. Am. J. Clin. Dermatol..

[12] Nam J.H., Yun Y., Kim H.S., Kim H.N., Jung H.J., Chang Y., Ryu S., Shin H., Kim H.L., Kim W.S. (2018). Rosacea and its association with enteral microbiota in Korean females. Exp. Dermatol..

[13] Chen Y.J., Lee W.H., Ho H.J., Tseng C.H., Wu C.Y. (2021). An altered fecal microbial profiling in rosacea patients compared to matched controls. J. Formos. Med. Assoc..

[14] Yun Y., Kim H.N., Chang Y., Lee Y., Ryu S., Shin H., Kim W.S., Kim H.L., Nam J.H. (2019). Characterization of the Blood Microbiota in Korean Females with Rosacea. Dermatology.

[15] Joura M.I., Jobbagy A., Dunai Z.A., Makra N., Banvolgyi A., Kiss N., Sardy M., Sandor S.E., Hollo P., Ostorhazi E. (2024). Characteristics of the Stool, Blood and Skin Microbiome in Rosacea Patients. Microorganisms.

[16] Jung W.H. (2023). Alteration in skin mycobiome due to atopic dermatitis and seborrheic dermatitis. Biophys. Rev..

[17] Szczepanska M., Blicharz L., Nowaczyk J., Makowska K., Goldust M., Waskiel-Burnat A., Czuwara J., Samochocki Z., Rudnicka L. (2022). The role of the cutaneous mycobiome in atopic dermatitis. J. Fungi..

[18] Wang R., Farhat M., Na J., Li R., Wu Y. (2020). Bacterial and fungal microbiome characterization in patients with rosacea and healthy controls. Br. J. Dermatol..

[19] Koike Y., Kuwatsuka S., Motooka D., Murota H. (2025). Dysbiosis of the human skin mycobiome in patients receiving systemic IL-23 inhibitors. Allergol. Int..

[20] Koike Y., Kuwatsuka S., Nishimoto K., Motooka D., Murota H. (2020). Skin mycobiome of psoriasis patients is retained during treatment with TNF and IL-17 inhibitors. Int. J. Mol. Sci..

[21] Tao R., Li R., Wang R. (2022). Dysbiosis of skin mycobiome in atopic dermatitis. Mycoses.

[22] Tao R., Wang R., Wan Z., Song Y., Wu Y., Li R. (2022). Ketoconazole 2% cream alters the skin fungal microbiome in seborrhoeic dermatitis: A cohort study. Clin. Exp. Dermatol..

[23] Wang X., Sun J., Zhang X., Chen W., Cao J., Hu H. (2024). Metagenomics reveals unique gut mycobiome biomarkers in psoriasis. Skin. Res. Technol..

[24] Chantanaskul T., Patumcharoenpol P., Roytrakul S., Kingkaw A., Vongsangnak W. (2024). Exploring protein functions of gut bacteriome and mycobiome in Thai infants associated with atopic dermatitis through metaproteomic and host interaction analysis. Int. J. Mol. Sci..

[25] Vanni P., Turunen J., Aijala V.K., Tapiainen V.V., Paalanne M., Pokka T., Paalanne N., Tejesvi M.V., Ruuska T.S. (2024). Gut mycobiome in atopic dermatitis and in overweight young children: A prospective cohort study in Finland. J. Fungi.

[26] Mok K., Suratanon N., Roytrakul S., Charoenlappanit S., Patumcharoenpol P., Chatchatee P., Vongsangnak W., Nakphaichit M. (2021). ITS2 sequencing and targeted meta-proteomics of infant gut mycobiome reveal the functional role of *Rhodotorula* sp. during atopic dermatitis manifestation. J. Fungi.

[27] Belvoncikova P., Splichalova P., Videnska P., Gardlik R. (2022). The human mycobiome: Colonization, composition and the role in health and disease. J. Fungi.

[28] Nash A.K., Auchtung T.A., Wong M.C., Smith D.P., Gesell J.R., Ross M.C., Stewart C.J., Metcalf G.A., Muzny D.M., Gibbs R.A. (2017). The gut mycobiome of the human microbiome project healthy cohort. Microbiome.

[29] Santus W., Devlin J.R., Behnsen J. (2021). Crossing kingdoms: How the mycobiota and fungal-bacterial interactions impact host health and disease. Infect. Immun..

[30] Xiong J., Chen S., Wang P., Chen A., Zheng Q., Cai T. (2023). Characterisation of the bacterial microbiome in patients with rosacea and healthy controls. Eur. J. Dermatol..

[31] Zhu W., Hamblin M.R., Wen X. (2023). Role of the skin microbiota and intestinal microbiome in rosacea. Front. Microbiol..

[32] Hu Q., Yu L., Zhai Q., Zhao J., Tian F. (2023). Anti-Inflammatory, barrier maintenance, and gut microbiome modulation effects of saccharomyces cerevisiae QHNLD8L1 on DSS-induced ulcerative colitis in mice. Int. J. Mol. Sci..

[33] Chiaro T.R., Soto R., Stephens W.Z., Kubinak J.L., Petersen C., Gogokhia L., Bell R., Delgado J.C., Cox J., Voth W. (2017). A member of the gut mycobiota modulates host purine metabolism exacerbating colitis in mice. Sci. Transl. Med..

[34] Sun S., Xu X., Liang L., Wang X., Bai X., Zhu L., He Q., Liang H., Xin X., Wang L. (2021). Lactic acid-producing probiotic saccharomyces cerevisiae attenuates ulcerative colitis via suppressing macrophage pyroptosis and modulating gut microbiota. Front. Immunol..

[35] Ramazzotti M., Stefanini I., Di Paola M., De Filippo C., Rizzetto L., Berna L., Dapporto L., Rivero D., Tocci N., Weil T. (2019). Population genomics reveals evolution and variation of Saccharomyces cerevisiae in the human and insects gut. Environ. Microbiol..

[36] Di Paola M., Rizzetto L., Stefanini I., Vitali F., Massi-Benedetti C., Tocci N., Romani L., Ramazzotti M., Lionetti P., De Filippo C. (2020). Comparative immunophenotyping of Saccharomyces cerevisiae and Candida spp. strains from Crohn’s disease patients and their interactions with the gut microbiome. J. Transl. Autoimmun..

[37] Itano A., Maslin D., Ramani K., Mehraei G., Carpenter N., Cormack T., Saghari M., Moerland M., Troy E., Caffry W. (2023). Clinical translation of anti-inflammatory effects of Prevotella histicola in Th1, Th2, and Th17 inflammation. Front. Med..

[38] Laigaard A., Krych L., Zachariassen L.F., Ellegaard-Jensen L., Nielsen D.S., Hansen A.K., Hansen C.H.F. (2020). Dietary prebiotics promote intestinal Prevotella in association with a low-responding phenotype in a murine oxazolone-induced model of atopic dermatitis. Sci. Rep..

[39] Zhong Y., Wang F., Meng X., Zhou L. (2024). The associations between gut microbiota and inflammatory skin diseases: A bi-directional two-sample Mendelian randomization study. Front. Immunol..

[40] Hertz S., Anderson J.M., Nielsen H.L., Schachtschneider C., McCauley K.E., Ozcam M., Larsen L., Lynch S.V., Nielsen H. (2024). Fecal microbiota is associated with extraintestinal manifestations in inflammatory bowel disease. Ann. Med..

